# Programmed Cell Death 2-Like (*Pdcd2l*) Is Required for Mouse Embryonic Development

**DOI:** 10.1534/g3.120.401714

**Published:** 2020-10-14

**Authors:** Brendan J. Houston, Manon S. Oud, Daniel M. Aguirre, D. Jo Merriner, Anne E. O’Connor, Ozlem Okutman, Stéphane Viville, Richard Burke, Joris A. Veltman, Moira K. O’Bryan

**Affiliations:** * School of Biological Sciences, Monash University, Clayton, Australia; † Department of Human Genetics, Radboud University Medical Center, Nijmegen, The Netherlands; ‡ Laboratoire de Diagnostic Génétique, UF3472-génétique de l’infertilité, Hôpitaux Universitaires de Strasbourg, Strasbourg, France; § Institut de Parasitologie et Pathologie Tropicale, EA 7292, Université de Strasbourg, 3 rue Koeberlé, Strasbourg, France; ** Centre for Life, Newcastle University, Newcastle Upon Tyne, UK; †† International Male Infertility Genomics Consortium

**Keywords:** *Pdcd2l*, embryonic development, acrosome, sperm function, male infertility

## Abstract

Globozoospermia is a rare form of male infertility where men produce round-headed sperm that are incapable of fertilizing an oocyte naturally. In a previous study where we undertook a whole exome screen to define novel genetic causes of globozoospermia, we identified homozygous mutations in the gene *PDCD2L*. Two brothers carried a p.(Leu225Val) variant predicted to introduce a novel splice donor site, thus presenting *PDCD2L* as a potential regulator of male fertility. In this study, we generated a *Pdcd2l* knockout mouse to test its role in male fertility. Contrary to the phenotype predicted from its testis-enriched expression pattern, *Pdcd2l* null mice died during embryogenesis. Specifically, we identified that *Pdcd2l* is essential for post-implantation embryonic development. *Pdcd2l^−/−^* embryos were resorbed at embryonic days 12.5-17.5 and no knockout pups were born, while adult heterozygous *Pdcd2l* males had comparable fertility to wildtype males. To specifically investigate the role of PDCD2L in germ cells, we employed *Drosophila melanogaster* as a model system. Consistent with the mouse data, global knockdown of *trus*, the fly ortholog of *PDCD2L*, resulted in lethality in flies at the third instar larval stage. However, germ cell-specific knockdown with two germ cell drivers did not affect male fertility. Collectively, these data suggest that *PDCD2L* is not essential for male fertility. By contrast, our results demonstrate an evolutionarily conserved role of *PDCD2L* in development.

Male infertility is a prevalent clinical presentation affecting at least 7% of men worldwide and is thought to have a large genetic component (Krausz and Riera-Escamilla 2018). Male fertility relies on the successful generation and maturation of sufficient numbers of functional sperm in order to reach and fertilize a receptive oocyte. While tmen suffering from poor sperm morphology and structure (teratozoospermia) are often capable of generating adequate numbers of sperm, these cells are often dysfunctional. Globozoospermia is an exceptionally damaging form of teratozoospermia, where men produce functionally incompetent sperm. These sperm possess rounded heads, with poorly compacted DNA and the absence of an acrosome. Globozoospermia is classified by severity, where type I signifies that 100% of sperm are acrosomeless, while type II signifies at least 50% of sperm are acrosomeless (Dam *et al.* 2007). The cause of this condition is largely based on defective proacrosomal vesicle formation or transport from the Golgi to the acrosome (Coutton *et al.* 2015; Pleuger *et al.* 2020). Ultimately, this results in the inability to penetrate the zona pellucida of the oocyte (Aitken *et al.* 1990).

Knockout mouse models for at least 48 independent genes have resulted in a globozoospermia phenotype. However, very few of these genes have also been defined as causative in men with globozoospermia (Coutton *et al.* 2015; Oud *et al.* 2020). We therefore set out to define novel genetic causes of globozoospermia using whole exome sequencing in a previous study (Oud *et al.* 2020). Two brothers of Turkish descent suffering from type II globozoospermia were studied by exome sequencing. Both brothers were homozygous for a frameshift variant in Gametogenetin (*GGN*) [NM_152657.3(*GGN*): c.1271del; p.(Gly424Alafs*65)] and a missense variant in Programmed cell death 2 like (*PDCD2L*) [NM_032346.1(PDCD2L):c.673T > G; p.(Leu225Val)] (Figure 1A). Although the amino acid change from leucine to valine on position 225 in *PDCD2L* was predicted to be neutral to protein function, the nucleotide change was predicted to introduce a novel splice donor site by several different algorithms.

**Figure 1:**
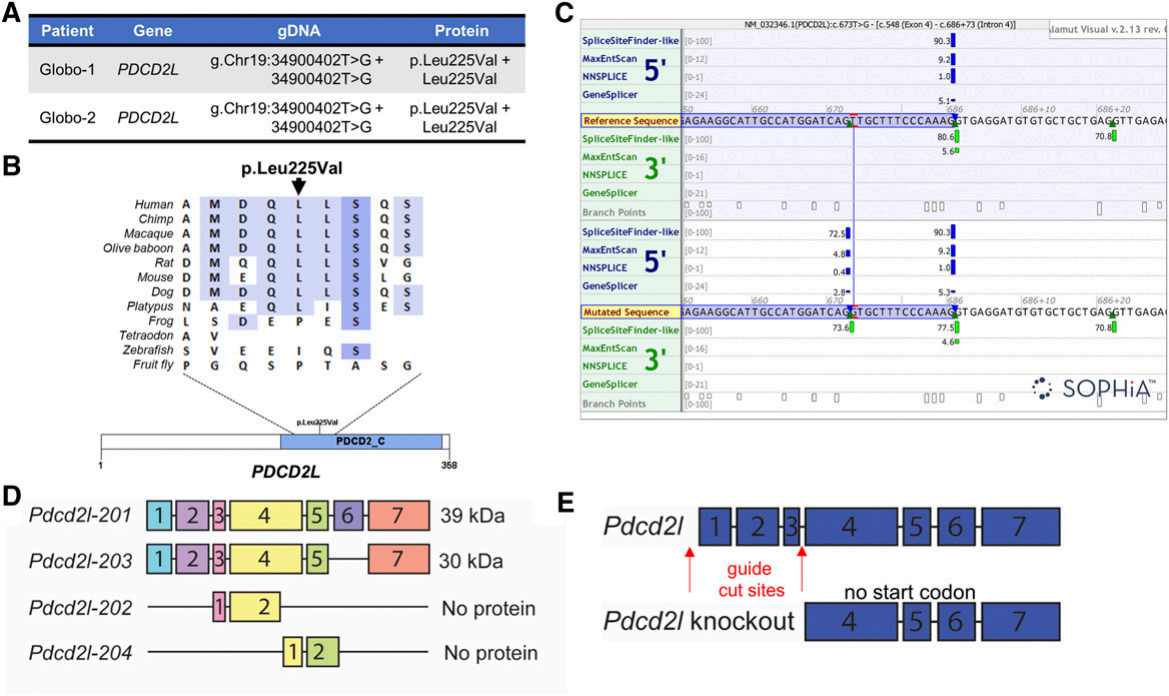
*PDCD2L* variant and knockout mouse strategy. A) Homozygous mutations within the *PDCD2L* gene were found in two brothers presenting with type II globozoospermia. B) The variant found in both brothers affects a moderately conserved leucine residue in the Programmed cell death protein 2, C-terminal domain (PDCD2_C; InterPro: IPR007320). C) Splice prediction models predict the introduction of a splice donor site in exon 4. The image depicts the border between exon 4 (highlighted by a blue box) and intron 4 in the wildtype (reference) *PDCD2L* sequence (top) and in the mutated situation (bottom). The site of the variant found in the two brothers is indicated in red. Hits from the four different splice prediction models (SpliceSiteFinder-like, MaxEntScan, NNSPLICE, GeneSplicer and Human Splicing Finder) are displayed as blue and green vertical bars for 5′ (donor) sites and 3′ (acceptor) sites, respectively. The computed score of each model is presented by the height of the green and blue vertical bars as a proportion of the maximum possible score and the individual scores are displayed left of the bars. The four splicing prediction models predict the introduction of a novel splice donor site within exon 4, without strongly disrupting the wildtype (reference) splice donor site. The ortholog alignment and splicing prediction was done by the Alamut Visual version 2.13 software package (http://www.interactive-biosoftware.com). D) Mouse *Pdcd2l* generates four transcripts, encoding two proteins at 39 and 30 kDa, as well as two untranslated transcripts. E) *Pdcd2l* knockout mouse strategy, with guide RNAs targeting exons 1-3.

Splicing of pre-mRNA products is dependent on the recognition of the 5′ and 3′ splice sites on the borders between exons and introns and at the branch site near the 3′ end of the intron. The splice sites are highly conserved and splicing at the donor site at the 5′ end of the intron happens between a G in the exon and an almost invariant sequence of GT in the intron (Zhang *et al.* 2018). The regions surrounding the canonical splice site sequence are less well conserved. Mutations affecting the highly or less highly conserved regions of splice sites are well known causes of disease (Wang and Cooper 2007). Alternatively, mutations can also activate cryptic splice sites that contain sequences similar to the consensus motifs of the canonical splice sites, or introduce novel splice sites (Anna and Monika 2018). The variant we identified in the two brothers with globozoospermia was predicted to create a novel splice donor site, which was slightly weaker than the wildtype splice site nearby but nevertheless potentially disrupts splicing of *PDCD2L*.

In mice, homozygous knockout of *Ggn* causes pre-implantation embryonic lethality which precluded the assessment of spermatozoa (Jamsai *et al.* 2013) and as such the role of the identified *GGN* variants in the etiology of globozoospermia remained unclear. As *PDCD2L* is highly expressed within the testis in humans (Fagerberg *et al.* 2014; NCBI Gene ID 84306) and is yet to be characterized in the context of male fertility, we generated a mouse knockout and also utilized fly knockdown models in this study to explore its role in male fertility and health broadly.

## Materials and methods

### Exome sequencing in two brothers with globozoospermia

Two brothers suffering from unexplained infertility gave informed consent for exome sequencing to elucidate the cause of infertility. Semen analysis was performed by the IVF clinics treating the patients. Exome sequencing was performed as described in Oud *et al.* (2020). This study was approved by the Comité de Protection de la Personne (CPP) at the University Hospital of Strasbourg, France.

### Splicing prediction methods

Splicing prediction based on the SpliceSiteFinder-like, MaxEntScan, NNSplice and GeneSplicer algorithms was done by the Alamut Visual version 2.13 software package (http://www.interactive-biosoftware.com).

### In-house database of Dutch fathers

As a control cohort, we used an anonymized dataset of 3,347 exomes of Dutch fathers. These are fathers of a child with a presumed genetic disorder such as intellectual disability referred for trio-based sequencing at Genome Diagnostics Nijmegen (https://www.genomediagnosticsnijmegen.nl/). These men may be carrier of a genetic disease, but their fertility is expected to be similar to unselected fathers.

### Animal ethics and generation of mutant mice

All mouse experiments adhered to animal ethics guidelines stated by the Australian National Health and Medical Research Council (NHMRC) and were approved by the Monash University Animal Ethics Committee (number BSCI/2017/31).

Guide RNA sequences (Table 1) were designed by the Australian Phenomics Network (Monash University, Australia) to target the removal of a region of *Pdcd2l* spanning exons 1 to 3 and including the start codon of the protein, using CRISPR/Cas9 technology (C57BL/6J mice). This gene modification resulted in a predicted complete loss-of-function mouse model, with no protein generated (Figure 1D). Founder mice were identified via Sanger sequencing and were supplied as heterozygotes, which were then crossed to generate presumptive knockouts. C57BL/6J wildtype littermates (*Pdcd2l^+/+^)* were utilized as wildtype controls in all experiments. All subsequent genotyping was performed by Transnetyx (Corvoda, USA) using primers listed in Table 2. Cryopreserved *Pdcd2l*^+/−^ is available upon request.

**Table 1:** Guide RNAs used to target excision of *Pdcd2l* exons 1-3

Guide RNAs	Upstream of exon 1	Downstream of exon 3
	AAAATAAAATAAGCCGGGTG	TCACTTTTATTCCACGTATA

**Table 2:** Primers used for genotyping *Pdcd2l* mutant mice

Genotyping primers	Forward	Reverse
*Pdcd2l* wildtype	GGGAATTGAGTTCAGGACCTCTTG	TCCAGCCTCCAAAAGCAATCTTT
*Pdcd2l* knockout	CCCGAGTCCTGGGATTAAAGG	GGCTTAAAGACACGTGCTACCA

### Expression of Pdcd2l

Human testis biopsies from healthy men were obtained with consent as described previously (Jamsai *et al.* 2008). Human and mouse whole testes were fixed in Bouin’s solution for 5 h at room temperature, transferred to 70% ethanol and processed into paraffin blocks using standard methods. 5 µm sections were cut and dried onto SuperFrost slides overnight at 37°. Testis sections were dewaxed and rehydrated using standard protocols, and antigen retrieval was performed as previously detailed (Hu *et al.* 2018). Testis sections were then blocked in CAS-block (Agilent, Santa Clara, CA, USA) for 30 min at room temperature. A PDCD2L mouse polyclonal antibody (SC-101251; Santa Cruz Biotechnology, USA) was added to sections (10 µg/ml in Dako Antibody diluent [Agilent]) and left on overnight at 4°. Testis sections were washed in PBS three times and a goat anti-mouse-488 secondary antibody (InVitrogen, Carlsbad, USA) was added (1/500 in 1 x PBS) for 1 h at room temperature. Sections were washed in PBS three times, then counterstained with 4 μM TO-PRO-3 nuclear stain (Thermo Fisher Scientific, Waltham, USA) for 30 min at room temperature. After washing in PBS once, each slide was mounted with Dako fluorescent mounting medium (Agilent).

Quantitative PCR (qPCR) was used to determine *Pdcd2l* expression levels within a host of murine organs, as previously described (Dunleavy *et al.* 2017). Isolated RNA was converted to cDNA using Superscript III (Invitrogen) and SYBR Green Master Mix (Thermo Fisher Scientific) was used for qPCR with primers shown in Table 3. Data on *Pdcd2l* expression at a single cell level within the testis was retrieved from a previously published mouse testis single cell RNA sequencing dataset (Jung *et al.* 2019)

**Table 3:** Primers used for quantitative PCR

qPCR primers	Forward	Reverse
Mouse		
*Pdcd2l*	CTGGCTGTTACCTGCCCTTC	GATCCACAACGCTGCCATAA
*Ppia*	CAGTGCTCAGAGCTCGAAAGTTT	TCTCCTTCGAGCTGTTTGCA
Fly		
*trus*	CACTGATCGTGCAGATGTACG	ATTCTGAGAGCAAACTGGGTTC
*RpL23*	GACAACACCGGAGCCAAGAACC	GTTTGCGCTGCCGAATAACCAC

### Assessment of embryonic lethality in mice

In order to assess the origin of the absence of knockout *Pdcd2l* mice, 10-12 week old male and 8-10 week old female heterozygous (het) *Pdcd2l* mice were housed for timed mating. After confirmation of mating by the presence of a copulatory plug, males were removed and pregnant female mice were allowed to advance to day 12.5 or 17.5 of pregnancy, at which stage they were culled. Females were dissected to access the uterus and determine the number of embryos, resorptions and total implantation sites for at least 3 independent females per time-point during gestation. Tail tips were collected from each fetus for genotyping, as described above.

### Phenotyping mice for male infertility

The fertility of male mice heterozygous for *Pdcd2l* was investigated as detailed previously (Borg *et al.* 2010). Briefly, mice were culled and weighed, and testes and epididymides were dissected out and weighed. One testis from each mouse was fixed and processed as detailed above, then stained with periodic acid-Schiff’s and hematoxylin reagents to investigate spermatogenesis histologically. The other testis was snap frozen on dry ice to investigate daily sperm production with the Triton-X-100 solubilization method (Cotton *et al.* 2006). Sperm were collected from the cauda epididymis via back-flushing (Smith *et al.* 2013) and transferred to MT6 medium. Sperm motility was assessed via computer assisted semen analysis as described previously (Gibbs *et al.* 2011) and an aliquot of the remaining sperm was dried onto a glass slide, fixed in 4% paraformaldehyde then stained with hematoxylin and eosin to investigate morphology.

### Knockdown of PDCD2L ortholog trus in Drosophila melanogaster

We sourced two independent RNA interference (RNAi) lines (22066 – line 1, 22067 – line 2) from the Vienna Drosophila Resource Center (VDRC; Vienna, Austria) and used these to investigate the effect of knockdown of *trus*, the fly *PDCD2L* ortholog, on male fertility and fly viability. We reared flies at 29° for all crosses. Flies were reared in plastic vials on standard food medium (Richards and Burke 2015). All fly lines used are detailed in Table 4.

**Table 4:** Fly lines used in this study and their genotypes

Line	Purpose	Genotype	Source
*w^1118^*	Wildtype control	w1118	Bloomington, line 3605
GFP RNAi	Non-specific RNAi control	w[*]; P{w[+mC]=UAS-GFP.dsRNA.R}143/CyO; Sb[1]/TM6B, Tb[1]	Bloomington, line 44412
Musashi (*msi*) RNAi	Infertile control	UAS-dicer; UAS-msi.dsRNA / [SM6B - TM6B]	Gary Hime lab, The University of Melbourne
*CLC-B* RNAi	Viable RNAi control	w1118; P{KK100840}VIE-260B	Vienna Drosophila Resource Centre, stock 103420
trus RNAi 1	Gene of interest knockdown	w1118; P{GD11610}v22066	Vienna Drosophila Resource Centre, stock 22066
trus RNAi 2	Gene of interest knockdown	w1118; P{GD11610}v22067	Vienna Drosophila Resource Centre, stock 22067
Nanos-Gal4; UAS-Gal4	Germ cell conditional driver, UAS drives maximal knockdown capacity	w[*];P{w[+mC]=UAS-GAL4}; P{w[+mC]=GAL4-nos.NGT}A	Gary Hime lab, The University of Melbourne
Bam-Gal4	Germ cell conditional driver	y[1] w[*] P{w[+mC]=bam-GAL4:VP16}1	Bloomington, line 80579
Tubulin-Gal4 with GFP balancer	Body-wide driver	Tub-GAL4/TM3, Sb, twi-GAL4, UAS-GFP	In-house, derived from Bloomington line 6663
Tubulin-Gal80ts; Tubulin-Gal4	Body-wide driver paired with temperature sensitive repressor	w[*]; P{w[+mC]=UAS-3xFLAG.dCas9.VPR}attP40, P{w[+mC]=tubP-GAL80[ts]}10; P{w[+mC]=tubP-GAL4}LL7/TM6B, Tb[1]	Bloomington, line 67065

To assess the role of *trus* in male fertility, we crossed the two RNAi lines to the Nanos-Gal4; UAS-Gal4 and Bam-Gal4 driver lines, which drive expression in early male germ cells within the *Drosophila* testis. We selected males from these crosses and fertility tested these alongside control males (*w^1118^* x Nanos-Gal4; UAS-Gal4 or *w^1118^* x Bam-Gal4) with virgin females from the wildtype *w^1118^* line (Bloomington Drosophila Stock Center [BL], Indiana, USA). To do so, individual males were placed with three virgin females for two days, and the number of pupae generated was counted after a further five days. A non-specific RNAi line, which targets GFP (BL-44412), was utilized as a second control line. A *musashi* RNAi line (Siddall *et al.* 2006) was used an infertile (Nanos) / sub-fertile (Bam) line. At least *n* = 10 individual males were fertility tested from each cross, except for Bam-Gal4 x GFP RNAi (*n* = 5).

To determine whether global loss of *trus* affected viability, we crossed the two *trus* RNAi lines to a ubiquitous Tubulin-Gal4 line carrying a GFP balancer (derived from BL-6663) and counted the number of eggs laid overnight in each vial. We then counted the total number of offspring, and percentage of GFP positive offspring at each developmental stage. Using these data, we were able to determine the stage at which lethality occurred (loss of GFP negative offspring). The *w^1118^* and *ClC-b* RNAi (VDRC, 103480) lines were used as non-lethal controls for the viability experiments.

To confirm that our *trus* RNAi lines resulted in specific knockdown, we crossed them to the Tubulin-Gal80; Tubulin-Gal4 driver and raised them at 18°. This allowed the Gal80 element to inhibit Gal4 and prevent lethality during development. We then collected adult males and maintained them at 29° for five days to induce Gal4 knockdown of *trus*. We extracted total mRNA using TRIzol reagent (Life Technologies, Scoresby, Australia) with the DirectZol RNA Miniprep kit (Zymo Research, Irvine, CA, USA). RNA was converted to cDNA using SuperScript III and *trus* RNA levels were measured via qPCR with SYBR green mastermix (Life Technologies) using a StudioQuant III system (Thermo Fisher Scientific, Scoresby, Australia). RNA extracted from stock TubGal80; TubGal4 flies was used as the wildtype control. Ct levels were normalized to the housekeeping gene Rpl23 (Gibbens *et al.* 2011). Primers used are shown in Table 3.

### Statistical analysis

Statistical significance was determined with Prism 8 (GraphPad, San Diego, USA) using a T-test, or ANOVA with post-hoc Dunnett’s test comparing to wildtype (control) samples. Significance was defined as *P* < 0.05.

### Data availability

All data are available within the manuscript and associated figures. All reagents and sources have been specified. Clinical genetic data were analyzed using the bioinformatics pipeline developed at the Department of Human Genetics Radboud Medical Centre, Nijmegen and further assessed with Alamut Visual version 2.13 software package (http://www.interactive-biosoftware.com).

## Results

Whole exome sequencing performed on infertile men with globozoospermia identified a point mutation in the gene *PDCD2L* (Figure 1A and B), as described in our previous work (Oud *et al.* 2019). This mutation was detected in two brothers as a homozygous Chr19(GRCh37):g.34900402T > G transversion and was predicted to result in an amino acid change of p.(Leu225Val). Of possibly greater functional relevance, the same variant was predicted to affect splicing of the RNA transcript (Figure 1C) and subsequently lead to a 14 bp deletion in the cDNA. Such a deletion would introduce a premature stop codon after three codons [p.(Leu225Profs*3)]. As premature stop codons early in a transcript often result in nonsense-mediated decay (Lindeboom *et al.* 2019), we also predicted that the variant would effectively lead to a null allele. The variant was absent in public variant databases including dbSNP and GnomAD. It was also absent from our control cohort of >3,000 Dutch fathers. DNA from the brothers’ parents and unaffected family members was not available, precluding further variant segregation studies.

In the mouse *Pdcd2l* has four transcript variants (Figure 1D), two of which do not generate a protein product (data sourced from Ensembl, ENSMUSG00000002635). The other two transcripts, denoted *Pdcd2l*-201 and *Pdcd2l*-203, are estimated to generate protein products of approximately 39 kD and 30 kD, respectively. To generate a full body mouse knockout, we elected to remove exons 1-3, including the start codon of the protein so that no protein would be generated (Figure 1E). Sequencing, then genotyping, of heterozygous mice confirmed successful deletion of the *Pdcd2l* gene.

### PDCD2L expression within the testis and other organs

**Figure 2:**
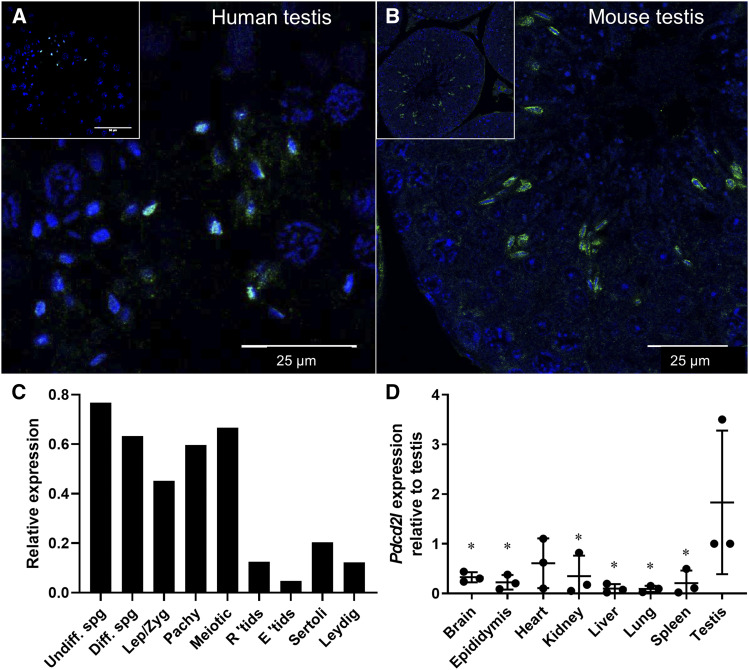
PDCD2L testis localization and tissue expression. PDCD2L immunolocalisation was explored in (A) human and (B) mouse testis. *Pdcd2l* transcript levels were retrieved from a mouse single cell testis sequencing database (C) and were measured via qPCR across major body organs (D). * *P* < 0.05 compared to testis. Undiff. spg = undifferentiated spermatogonia, Diff. spg = differentiated spermatogonia, Lep/Zyg = leptotene/zygotene spermatocytes, Pachy = pachytene spermatocytes, R ‘tids = round spermatids, E ‘tids = elongating spermatids.

We defined the localization of PDCD2L in human and mouse testis and assessed its potential role in spermatogenesis (Figure 2) via immunofluorescence using a PDCD2L antibody. PDCD2L was localized to the elongating spermatid population in both human (Figure 2A) and mouse (Figure 2B) testes. Specifically, PDCD2L localized to the developing acrosome. We next investigated *Pdcd2l* transcript expression using published testis single cell sequencing data (Figure 2C, Jung *et al.* 2019). Here, it was seen that *Pdcd2l* expression was highest in spermatogonia and enriched in spermatocytes just prior to spermatid formation and differentiation. We also investigated the expression of *Pdcd2l* across major organs (Figure 2D), which revealed a testis enrichment, and comparatively lower expression in the brain, epididymis, kidney, liver, lung, and spleen (*P* < 0.05).

### Knockout of Pdcd2l causes embryonic lethality

**Figure 3:**
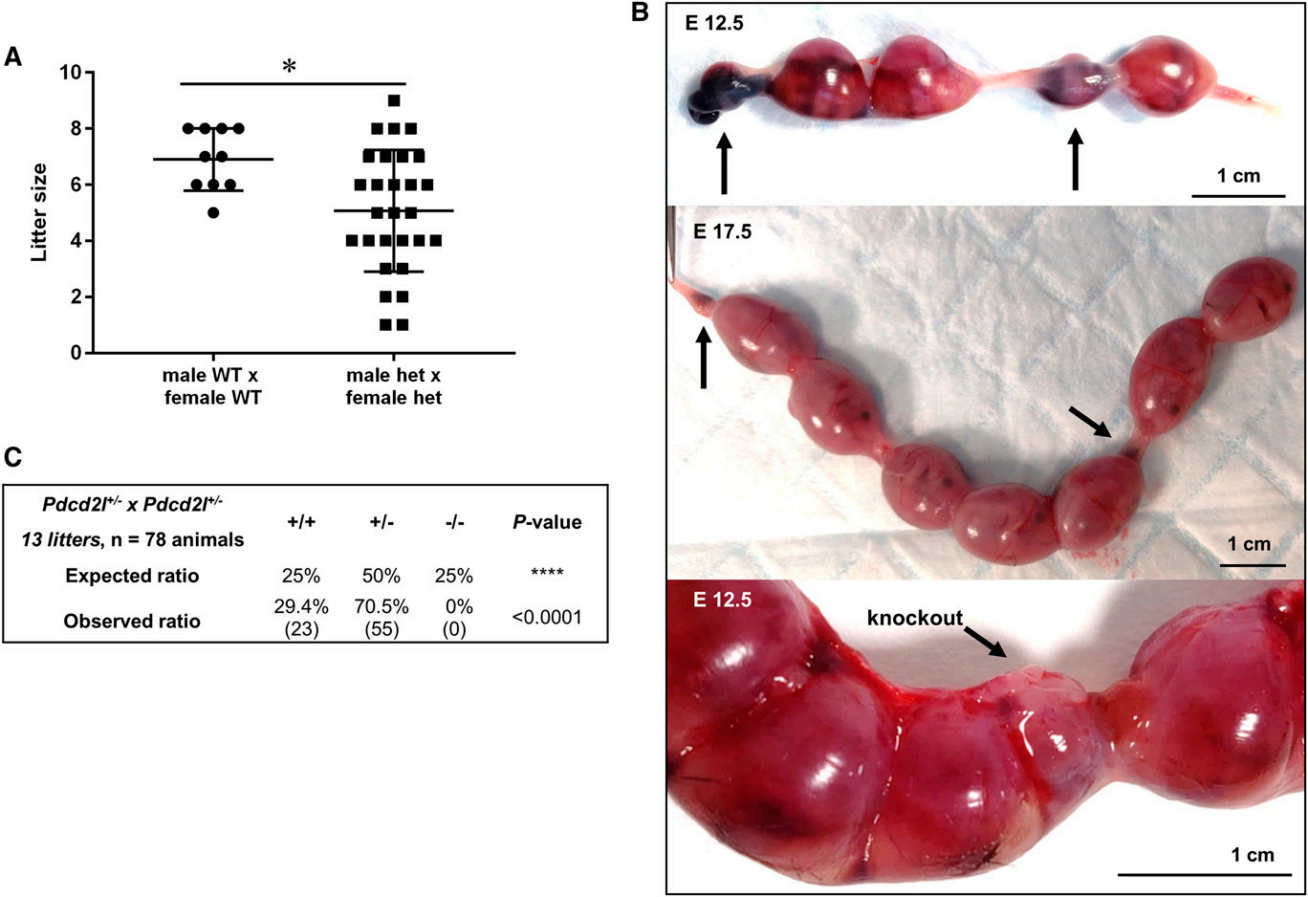
Loss of *Pdcd2l* causes embryonic death in mice. A) Litter sizes of wildtype and heterozygous matings. B) Embryo resorptions in mouse uteri from heterozygous *Pdcd2l* matings, at embryonic days [E] 12.5 and 17.5. Arrows point to embryo resorptions and in the bottom panel indicate a small embryo that was genotyped as a knockout. C) Analysis of genotype ratios from heterozygous matings in comparison to the anticipated Mendelian inheritance of 1:2:1 wildtype:heterozygous:knockout pups.

Mating between heterozygous male and female mice (Figure 3A) failed to generate any knockout pups (n = 28 litters from 11 individual pair matings). In complement, the average litter size of heterozygous matings was significantly smaller than wildtype matings at ∼5.07 *vs.* 6.90 pups (*P* = 0.016), respectively (Figure 3A). As such, we investigated whether the loss of *Pdcd2l* led to embryonic lethality (Figure 3B). To do this, heterozygous mice were mated and plug checked to determine day 0 of pregnancy. The number of implantation sites and embryo resorptions was investigated in the uteri of females at days 12.5 and 17.5 of pregnancy, to represent mid and late gestation (Figure 3B). We identified embryo resorptions in the uteri of females at both day 12.5 and 17.5 of pregnancy. All carried embryo resorptions, except one female at day 12.5. Another female at day 12.5 carried a small embryo that genotyped as knockout (Figure 3B, bottom panel). No knockout pups were born from heterozygous matings (Figure 3C) (*P* < 0.0001).

### Fertility of heterozygous Pdcd2l males

**Figure 4:**
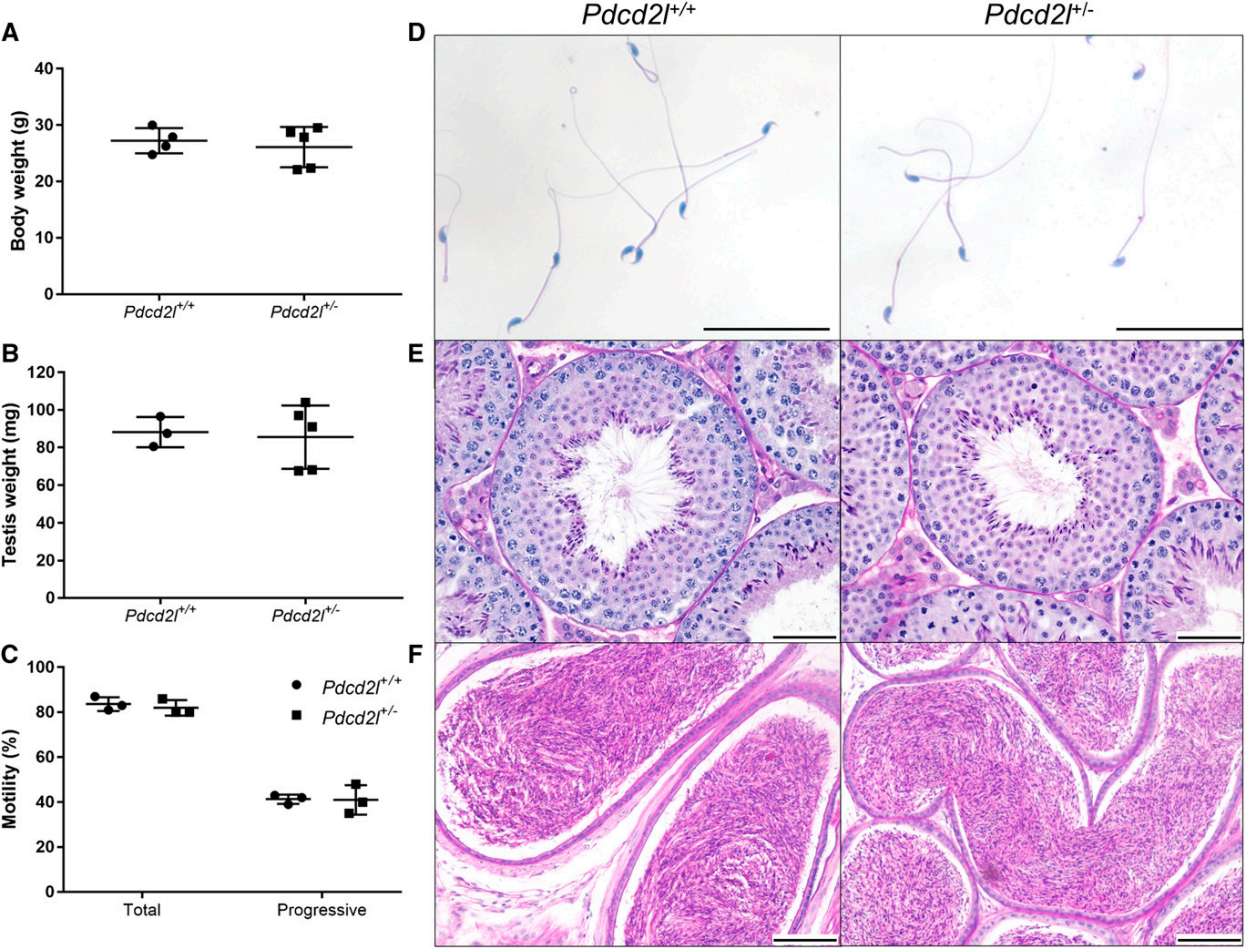
Fertility examination of heterozygous *Pdcd2l* male mice in comparison to wildtype males. Routine analyses were performed, comparing the following across genotypes: (A) body weight, (B) testis weight, (C) sperm motility analysis, (D) sperm morphology, (E) testis histology and (F) epididymal histology.

To assess whether heterozygous *Pdcd2l* males possessed similar levels of fertility to wildtype counterparts, we investigated sperm production, morphology and motility, and testicular histology (Figure 4). *Pdcd2l^+/−^* males had similar body (Figure 4A) and testis (Figure 4B) weights to wildtype males, and their sperm demonstrated equal levels of total and progressive motility (Figure 4C). Further, *Pdcd2l^+/−^* males generated morphologically normal sperm (Figure 4D) and no overt effects to spermatogenesis (Figure 4E) or epididymal histology (Figure 4F) were observed.

### Drosophila trus RNA interference screen

**Figure 5:**
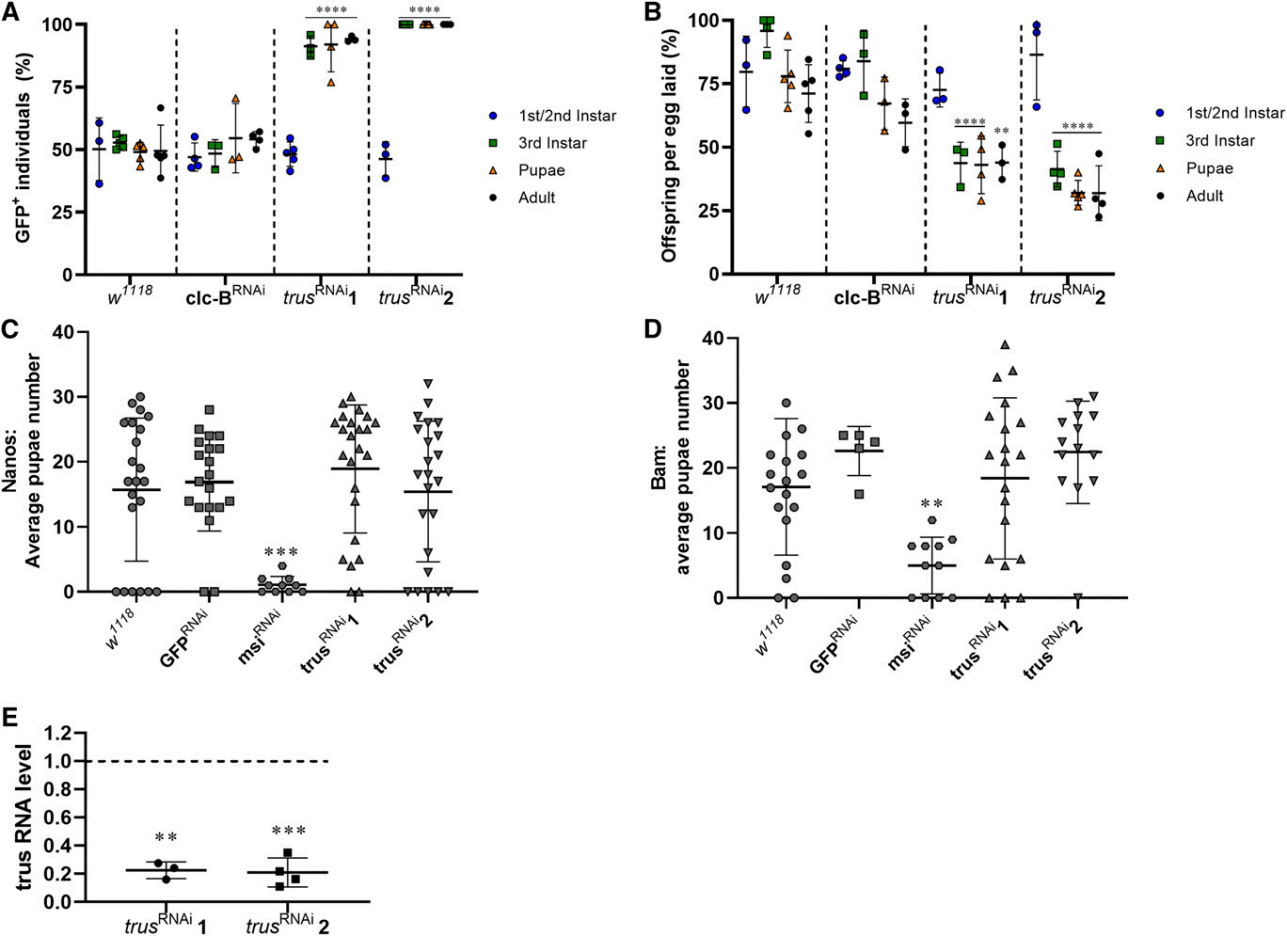
Ubiquitous knockdown of the *PDCD2L* fly ortholog *trus* causes lethality but does not affect germ cell specific development. A) Developmental rate and B) GFP+ rate (RNAi element absent) of individuals from each line crossed to Tub-Gal4 with GFP balancer. Fertility was tested in males with germ cell-depleted *trus* levels, using Nanos (C) and Bam (D) drivers, through counting pupae generated after mating with *w^1118^* females. Each data point represents a single vial counted. ** *P* < 0.01, *** *P* < 0.001, **** *P* < 0.0001 compared to *w^1118^* at corresponding developmental stage. E) Knockdown efficiency of each *trus* RNAi line as measured by quantitative PCR, normalized to *RpL23*. ** *P* < 0.01, *** *P* < 0.001 compared to wildtype.

As we were unable to generate viable knockout *Pdcd2l* mice, we utilized RNA interference in *Drosophila melanogaster* to knockdown transcript levels of the fly *Pdcd2l* ortholog *(**trus**)* (Figure 5). First, we sourced two commercially available RNAi lines for *trus* and crossed them to a ubiquitously expressed Tubulin-Gal4 driver (carrying a GFP balancer) to induce global knockdown of *trus* (Figure 5A, B). We also crossed Tubulin-Gal4; GFP balancer to control *w^1118^* and a non-lethal RNAi line for *ClC-b*. The total offspring:egg ratio and percentage of GFP^-^ offspring (RNAi containing) were scored at each stage. In agreement with lethality data in knockout mice, ubiquitous knockdown of *trus* in flies resulted in significant lethality at the third instar larval stage of both knockdown lines (*P* < 0.0001 compared to *w^1118^*) (Figure 5A), as was highlighted by a reduction in the ratio of GFP^-^ larvae compared to control crosses. Specifically, we observed 100% lethality at the third instar larval stage for the *trus* RNAi-2 line, and on average 82% lethality for the *trus* RNAi-1 line (91% GFP rate with an expected 50% GFP rate in a non-lethal cross). No GFP^-^ individuals were recorded for *trus* RNAi-2 cross beyond the third instar stage and the lethality rate did not increase for individuals in the *trus* RNAi-1 cross beyond the third instar stage. In accordance, we observed a significant reduction in the number of viable offspring in both RNAi lines in comparison to both control lines from the third-instar stage (Figure 5B, *P* < 0.0001). The *ClC-b* RNAi cross displayed a similar incidence of GFP^-^ individuals and offspring development rates to the *w^1118^* cross at all developmental stages.

Finally, we crossed the two *trus* RNAi lines to a Nanos driver (Nanos-Gal4; UAS-Gal4) and a Bam driver (Bam-Gal4) to generate germ cell-specific knockdowns of *trus*, allowing us to explore its role in male fertility (Siddall and Hime 2017). We investigated the number of pupae generated by each male with a mating assay (Figures 5C, D). Equivalent levels of fertility were observed between control knockdown males (*w^1118^* and non-specific [GFP] RNAi), and males with germ cell knockdowns of *trus* using both the Nanos-Gal4 (Figure 5C) and Bam-Gal4 (Figure 5D) drivers. These data reveal that *Pdcd2l* does not play an essential role in fly spermatogenesis and male fertility.

To quantify the level of *trus* knockdown, we crossed both RNAi lines to a Tubulin-Gal80; Tubulin-Gal4 line and shifted the flies from 18° to 29° once the flies reached adulthood, then extracted mRNA five days later. We then performed qPCR to measure *trus* RNA levels (Figure 5E). We found, on average, a 77.5% reduction in *trus* expression in the RNAi-1 line and a 79% reduction in the RNAi-2 line, thus confirming the validity of the knockdown strategy.

## Discussion

Within this study we demonstrated that *Pdcd2l* plays an evolutionarily conserved and essential role in embryonic / early development. While the definitive reason for lethality in loss-of-function animals is unclear, *in situ* hybridization for *Pdcd2l* performed on mouse embryos at e10.5 by Bolkan (2008) revealed specific expression within the embryonic brain, highlighting impaired brain development as a potential cause of death. PDCD2L has also been hypothesized to play an important role in supporting maturation of 40S ribosomal units, and it may therefore be systemically essential for health across multiple organs (Landry-Voyer *et al.* 2016). Furthermore, and supporting the results of our study, Bolkan (2008) also documented lethality of *trus* depleted flies.

Globozoospermia is a rare and severe infertility phenotype and frequently has a genetic etiology (Koscinski *et al.* 2011, Perrin *et al.* 2013). In a previous study we identified candidate infertility-causing variants in *PDCD2L* in two brothers with globozoospermia (Oud *et al.* 2020). *PDCD2L* shows a highly enriched testis expression pattern and is localized to the developing acrosome. Contrary to expectations, the loss of *Pdcd2l* expression in the mouse resulted in embryonic lethality during mid-gestation, and thus precluded us from testing the role of PDCD2L in acrosome formation and sperm function. As such, we generated a germ cell *trus* knockdown and determined that it did not impact male fertility in flies.

Our results strongly support an essential role for *PDCD2L* in development/health, but the two brothers in our screen who carried mutations in *PDCD2L* were alive and seeking fertility treatment. This suggests that either the variant found in these brothers did not induce a significant loss of function, or that *PDCD2L* function is redundant in humans. Although the *PDCD2L* variant identified in the brothers was anticipated to introduce a novel splice site, the *de novo* splice site is predicted to be weaker than the wildtype splice donor site, and thus may not be utilized (Figure 1C). Consistent with this interpretation, a recently released splice prediction algorithm based on machine leaning principles (Jaganathan *et al.* 2019) predicted the variant was unlikely to lead to aberrant splicing (∆ Score donor gain = 0.04, ∆ Score donor loss 0.01; high precision cut-off for being splice-altering = ∆ Score >0.8 [range 0-1]). It is also possible that this mutation resulted in a novel gain-of-function allele and that our mouse and fly knockout/knockdown approaches do not accurately model the biological effect of the variant in humans.

Similar to variants affecting the canonical splice site, variants that introduce a premature stop codon (nonsense) and variants that shift the reported transcriptional frame (frameshift) are considered as loss-of-function variation (Karczewski *et al.* 2020). To date, no individuals with other homozygous *PDCD2L* loss-of-function variants have been described in the population database gnomAD or in our in-house exome database of 3,347 fathers (materials and methods). In these databases we did however identify 89 and 5 heterozygous carriers, respectively, of *PDCD2L* LoF variants, demonstrating that heterozygous loss-of-function of *PDCD2L* is not lethal and does not cause sterility. Based on these databases alone, it is currently impossible to predict whether homozygous knockout of *PDCD2L* in human causes embryonic lethality.

In conclusion, our data suggest that *PDCD2L* is not essential for male fertility in the fly and potentially not in mice. Our results do, however, demonstrate an evolutionarily conserved role of PDCD2L in development.

## References

[bib1] Aitken R. J., Kerr L., Bolton V., and Hargreave T., 1990 Analysis of sperm function in globozoospermia: implications for the mechanism of sperm-zona interaction. Fertil. Steril. 54: 701–707. 10.1016/S0015-0282(16)53833-3 2209893

[bib2] Anna A., and Monika G., 2018 Splicing mutations in human genetic disorders: examples, detection, and confirmation. J Appl Genet. 59: 253–268. 10.1007/s13353-018-0444-7 29680930 PMC6060985

[bib3] Bolkan, B., 2008. An analysis of proposed mitotic defects in the toys are us mutant and Drosophila hybrids. PhD thesis, Cornell University, Ithaca, NY.

[bib28] Borg C. L., Wolfski K. M., Gibbs G. M., O’Bryan M. K., 2010 Phenotyping male infertility in the mouse: how to get the most out of a ‘non-performer’. Hum. Reprod. *Update*. 16: 205–224. 10.1093/humupd/dmp032 PMC2816191 19758979

[bib4] Cotton L. G. M., Gibbs L. G., Sanchez-Partida J. R., Morrison D. M., de Kretser D. M., 2006 FGFR-1 [corrected] signaling is involved in spermiogenesis and sperm capacitation. J. Cell Sci. 119: 75–84. 10.1242/jcs.02704 16352663

[bib5] Coutton C., Escoffier J., Martinez G., Arnoult C., and Ray P. F., 2015 Teratozoospermia: spotlight on the main genetic actors in the human. Hum. Reprod. Update 21: 455–485. 10.1093/humupd/dmv020 25888788

[bib6] Dam A. H., Feenstra I., Westphal J. R., Ramos L., van Golde R. J., 2007 Globozoospermia revisited. Hum. Reprod. Update 13: 63–75. 10.1093/humupd/dml047 17008355

[bib7] Dunleavy J. E. M., Okuda H., O’Connor A. E., Merriner D. J., O’Donnell L., 2017 Katanin-like 2 (KATNAL2) functions in multiple aspects of haploid male germ cell development in the mouse. PLoS Genet. 13: e1007078 10.1371/journal.pgen.1007078 29136647 PMC5705150

[bib8] Fagerberg L., Hallstrom B. M., Oksvold P., Kampf C., Djureinovic D., 2014 Analysis of the human tissue-specific expression by genome-wide integration of transcriptomics and antibody-based proteomics. Mol. Cell. Proteomics 13: 397–406. 10.1074/mcp.M113.035600 24309898 PMC3916642

[bib29] Hu J., Merriner D. J., O'Connor A. E., Houston B. J., Furic L., 2018 Epididymal cysteine-rich secretory proteins are required for epididymal sperm maturation and optimal sperm function. Mol. Hum. Reprod. 24: 111–112. 29361143 10.1093/molehr/gay001

[bib9] Gibbens Y. Y., Warren J. T., Gilbert L. I., and O’Connor M. B., 2011 Neuroendocrine regulation of Drosophila metamorphosis requires TGFbeta/Activin signaling. Development 138: 2693–2703. https://doi.org10.1242/dev.063412 21613324 10.1242/dev.063412 PMC3109597

[bib10] Gibbs G. M., Orta G., Reddy T., Koppers A. J., Martinez-Lopez P., 2011 Cysteine-rich secretory protein 4 is an inhibitor of transient receptor potential M8 with a role in establishing sperm function. Proc. Natl. Acad. Sci. USA 108: 7034–7039. 10.1073/pnas.1015935108 21482758 PMC3084142

[bib11] Jaganathan K., Kyriazopoulou Panagiotopoulou S., McRae J. F., Darbandi S. F., Knowles D., , 2019 Predicting Splicing from Primary Sequence with Deep Learning. Cell 176: 535–548.e24. 10.1016/j.cell.2018.12.015 30661751

[bib12] Jamsai D., O’Connor A. E., DeBoer K. D., Clark B., Smith S. J., 2013 Loss of GGN leads to pre-implantation embryonic lethality and compromised male meiotic DNA double strand break repair in the mouse. PLoS One 8: e56955 10.1371/journal.pone.0056955 23451117 PMC3579931

[bib13] Jamsai D., Reilly A., Smith S. J., Gibbs G. M., Baker H. W. G., 2008 Polymorphisms in the human cysteine-rich secretory protein 2 (CRISP2) gene in Australian men. Hum. Reprod. 23: 2151–2159. 10.1093/humrep/den191 18550510

[bib14] Jung M., Wells D., Rusch J., Ahmad S., Marchini J., 2019 Unified single-cell analysis of testis gene regulation and pathology in five mouse strains. Elife. 8: e43966 10.7554/eLife.43966 31237565 PMC6615865

[bib15] Karczewski K. J., Francioli L. C., Tiao G., Cummings B. B., Alfoldi J., 2020 The mutational constraint spectrum quantified from variation in 141,456 humans. Nature 581: 434–443. 10.1038/s41586-020-2308-7 32461654 PMC7334197

[bib16] Koscinski I., Elinati E., Fossard C., Redin C., Muller J., 2011 DPY19L2 deletion as a major cause of globozoospermia. Am. J. Hum. Genet. 88: 344–350. 10.1016/j.ajhg.2011.01.018 21397063 PMC3059416

[bib17] Krausz C., and Riera-Escamilla A., 2018 Genetics of male infertility. Nat. Rev. Urol. 15: 369–384. 10.1038/s41585-018-0003-3 29622783

[bib18] Landry-Voyer A. M., Bilodeau S., Bergeron D., Dionne K. L., Port S. A., 2016 Human PDCD2L Is an Export Substrate of CRM1 That Associates with 40S Ribosomal Subunit Precursors. Mol. Cell. Biol. 36: 3019–3032. 10.1128/MCB.00303-16 27697862 PMC5126290

[bib19] Lindeboom R. G. H., Vermeulen M., Lehner B., and Supek F., 2019 The impact of nonsense-mediated mRNA decay on genetic disease, gene editing and cancer immunotherapy. Nat Genet. 51: 1645–1651. 10.1038/s41588-019-0517-5 31659324 PMC6858879

[bib20] Oud M. S., Okutman O., Hendricks L. A. J., de Vries P. F., Houston B. J., 2020 Exome sequencing reveals novel causes as well as new candidate genes for human globozoospermia. Hum. Reprod. 35: 240–252. 10.1093/humrep/dez246 31985809 PMC6993856

[bib21] Perrin A., Coat C., Nguyen M. H., Talagas M., Morel F., 2013 Molecular cytogenetic and genetic aspects of globozoospermia: a review. Andrologia 45: 1–9. 10.1111/j.1439-0272.2012.01308.x 22571172

[bib22] Pleuger C., Lehti M. S., Dunleavy J. E., Fietz D., and O’Bryan M. K., 2020 Haploid male germ cells-the Grand Central Station of protein transport. Hum Reprod Update 26: 474–500. 10.1093/humupd/dmaa004 32318721

[bib23] Richards C. D., and Burke R., 2015 Local and systemic effects of targeted zinc redistribution in Drosophila neuronal and gastrointestinal tissues. Biometals 28: 967–974. 10.1007/s10534-015-9881-5 26411574

[bib30] Siddall N. A., McLaughlin E. A., Marriner N. L., Hime G. R., 2006 The RNA-binding protein Musashi is required intrinsically to maintain stem cell identity. PNAS. 103: 8402–8407. 10.1073/pnas.0600906103 16717192 PMC1570104

[bib24] Siddall N. A., and Hime G. R., 2017 A Drosophila toolkit for defining gene function in spermatogenesis. Reproduction 153: R121–R132. 10.1530/REP-16-0347 28073824

[bib25] Smith T. B., De Iuliis G. N., Lord T., and Aitken R. J., 2013 The senescence-accelerated mouse prone 8 as a model for oxidative stress and impaired DNA repair in the male germ line. Reproduction 146: 253–262. 10.1530/REP-13-0186 23813448

[bib26] Wang G. S., and Cooper T. A., 2007 Splicing in disease: disruption of the splicing code and the decoding machinery. Nat Rev Genet. 8: 749–761. 10.1038/nrg2164 17726481

[bib27] Zhang S., Samocha K. E., Rivas M. A., Karczewski K. J., Daly E., 2018 Base-specific mutational intolerance near splice sites clarifies the role of nonessential splice nucleotides. Genome Res. 28: 968–974. 10.1101/gr.231902.117 29858273 PMC6028136

